# SM-CycleGAN: crop image data enhancement method based on self-attention mechanism CycleGAN

**DOI:** 10.1038/s41598-024-59918-3

**Published:** 2024-04-23

**Authors:** Dian Liu, Yang Cao, Jing Yang, Jianyu Wei, Jili Zhang, Chenglin Rao, Banghong Wu, Dabin Zhang

**Affiliations:** 1 School of Mechanical Engineering, Guizhou University, Guiyang, 550025 China; 2 China Tobacco Guangxi Industrial Co., Ltd, Nanning, 530000 China; 3 State Key Laboratory of Public Big Data, Guizhou University, Guiyang, 550025 China

**Keywords:** Self-attention mechanism, Cyclic consistency adversarial network, Tea disease, Tobacco roasting, PSNR, SSIM, Mechanical engineering, Computer science

## Abstract

Crop disease detection and crop baking stage judgement require large image data to improve accuracy. However, the existing crop disease image datasets have high asymmetry, and the poor baking environment leads to image acquisition difficulties and colour distortion. Therefore, we explore the potential of the self-attention mechanism on crop image datasets and propose an innovative crop image data-enhancement method for recurrent generative adversarial networks (GANs) fused with the self-attention mechanism to significantly enhance the perception and information capture capabilities of recurrent GANs. By introducing the self-attention mechanism module, the cycle-consistent GAN (CycleGAN) is more adept at capturing the internal correlations and dependencies of image data, thus more effectively capturing the critical information among image data. Furthermore, we propose a new enhanced loss function for crop image data to optimise the model performance and meet specific task requirements. We further investigate crop image data enhancement in different contexts to validate the performance and stability of the model. The experimental results show that, the peak signal-to-noise ratio of the SM-CycleGAN for tobacco images and tea leaf disease images are improved by 2.13% and 3.55%, and the structural similarity index measure is improved by 1.16% and 2.48% compared to CycleGAN, respectively.

## Introduction

Cash crops (e.g. tobacco, tea) are critical in agricultural development. During crop roasting (e.g. tobacco), the colour of the leaf is the most intuitive observational guide to the maturity of the tobacco roast and is defined as a maturity index in determining, for example, the harvest time. As the maturation process changes, the most prominent feature is the change in leaf colour to yellow. However, the harsh environment of tobacco roasting makes image acquisition challenging, and images are prone to colour distortion. Crop leaf (e.g. tea) diseases are severe problems faced by the agro-industry, and tea disease recognition and detection require large data as a dataset to improve the recognition accuracy. However, the image data for tea diseases are small and challenging to collect. Therefore, crop image distortion recovery and image data augmentation can be performed using image data-enhancement methods.

Image data-enhancement methods are widely used to solve problems of insufficient datasets and image restoration and can be broadly categorised into traditional-based and deep-learning-based methods. Traditional-based methods are typically based on the direct manipulation of image pixel values and mathematical transformations, such as spatial transformation-based image flipping, cropping, rotating, scaling, morphing^1^, filtering-based methods^2^, and multi-sample synthesis techniques such as sample pairing. Image enhancement based on deep-learning methods uses deep-learning network technology to process the original image, learn image features and the relationship between them and process them to improve the image quality and visual effects, such as generative adversarial networks (GANs)^3^ and image restoration^4^. Although traditional digital enhancement methods are straightforward, intuitive and interpretable, they have poor generalisation and are challenging to generalise to diverse image data. However, deep-learning-based image data-enhancement methods can adapt to different tasks and data distributions, perform image data enhancement on diverse image data and are more effective under large-scale data. In recent years, deep learning has made breakthrough progress in crop image data enhancement (CIDE), which has a fundamental role in practical applications, such as plant classification data augmentation^5,6^ and plant disease identification data enhancement and detection^7,8^, and has received increasing attention. It can extract and learn high-level features from images, making it is suitable for extracting visual features of plant leaves and stems. GAN learns from the feature distributions of the original images and generates images with similar distributions, which can provide more feature information for classification models. Despite the many advantages of GAN, the training process is slow to converge and prone to crashes.

The cycle-consistent GAN (CycleGAN) is a derivative model of GAN, an unsupervised learning method^9^. Its core idea is derived from GANs and by introducing the cyclic consistency loss. It can realise unsupervised image transformation from one domain to another and simultaneously realise mutual data transformation in two image domains. The CycleGAN models have been used for leaf disease identification^10,11^, citrus-greening disease generation and recognition^12^, plant image synthesis generation^13^ and leaf disease sample amplification and classification^5,6^. Although CycleGAN has made significant progress, its images are too similar, lack diversity and its unsupervised nature makes it challenging to provide fine control over the generated images. The self-attention mechanism is used to process sequential data and is widely used in deep-learning models, such as crop leaf disease identification and detection^14–16^, rice pest detection^17^ and crop classification and identification^18,19^. Its introduction helps improve the performance of the model, allows the model to capture global and local image information better and helps generate higher quality and a greater number of images. Therefore, the self-attention mechanism is added to the CycleGAN model to improve the quality of image recovery and data diversity.

Based on the above, we propose a new CIDE method based on the CycleGAN with the self-attention mechanism. Our method introduces CycleGAN as the primary framework of the network. It adds self-attention to the generator and discriminator to help the network focus on more critical regions in the image and extract essential feature information to improve image data diversity. Furthermore, SM-CycleGAN can be used for tobacco drying, tea disease data enhancement and image data enhancement of other economic crops. The primary contributions of this paper are the following.

1. We have proposed a new SM-CycleGAN crop image-data-augmentation method that introduces the self-attention mechanism into the generator and discriminator of the main network framework CycleGAN. By helping the CycleGAN network focus on important areas in the image, we can extract important feature information of the image and reduce the interference of noise. Thus, the dependence and connection between normal crop images and distorted (disease) crop images are learned, improving the diversity of image data.
2. We have added the self-attention mechanism to the backbone network of CycleGAN to strengthen the learning of image features and the relationship between images in the crop image data enhancement task. By accurately capturing the key feature information between the original image and the target image, the accuracy and performance of the image data enhancement task and the adaptive mapping ability of the model are improved. Meanwhile, based on the original CycleGAN loss and the loss of self-attention mechanism, a new CIDE loss function is designed to achieve more realistic image-data enhancement.
3. We have conducted a comprehensive experimental evaluation of SM-CycleGAN in the scenarios of tobacco-leaf image-distortion restoration and tea disease image-data enhancement, and we conducted ablation experiments and comparison experiments with baseline methods and existing advanced methods. The experimental results show that SM-CycleGAN have a significant effect on image processing. Moreover, in the future, SM-CycleGAN can not only be used for image-data enhancement of tobacco-leaf drying and tea disease but also for image-data enhancement of other cash crops.

The rest of paper is organised as follows. In “Related work” section presents research-related work. In “Methods” section proposes an SM-CycleGAN-based image data-enhancement method and network for cash crops. In “Isolated crop image online acquisition equipment” section demonstrates an online image acquisition device for isolated crop drying. In “Experimentation and analysis” section, experiments and analysis are performed. The conclusions are presented in “Conclusion” section.

## Related work

### Traditional image data processing methods

In recent years, traditional image data processing methods have achieved remarkable results in crop leaf disease image data enhancement and colour distortion recovery. Hu, GS^1^ proposed a geometric semantic segmentation method for natural scene images of tea leaves based on the discriminative pyramid network, which uses techniques such as image flipping, translating, mirroring and random scaling to enhance the training samples. It adopts local histogram equalisation techniques to reduce the effect of uneven illumination on segmentation. To improve and enhance the richness and diversity of the tea training samples, Bao^20^ used offline data-enhancement techniques to pan, rotate by 30° and horizontally flip the original training images before training and detecting the network to obtain rich angular information about the target. Bao^21^ expanded the number of training and validation images by horizontally flipping, rotating and translating wheat images. Arun Pandian et al.^22^ developed a dataset of augmented plant leaf diseases using conventional image processing, such as image flipping, cropping, rotation, colour transformation, principal components analysis (PCA) colour enhancement and noise injection techniques. While photographing leaf samples at various times during tobacco roasting, Odabas, Mehmet Serhat^23^ used the X-rite colour checker for colour correction to eliminate the negative effect of ambient light. However, all the above conventional methods for image data enhancement require manual extraction of lesion features or colour features and are limited in their effectiveness in processing similar disease features or colour features. Furthermore, such methods cannot increase the diversity of images and, thus, cannot solve the diversity problem.

### Machine-learning-based image data processing methods

With the continuous development of machine learning, some researchers have applied convolutional neural networks (CNNs) to crops such as wheat^24^, cucumber^25^, cereal^26–28^ and apple leaf diseases^29^. Experiments with different crop datasets show that deep-learning methods are significantly better than traditional machine-learning methods. It is well known that deep-learning methods require extensive data. However, collecting sufficient and usable datasets is unreliable and time-consuming. In agriculture, data availability faces significant challenges due to the collection of light conditions and collection equipment, and the dependence on the annotation of the dataset size increases. Therefore, synthetic datasets have been investigated in recent years. Data expansion aims to increase the dataset size^30^, which is widely used in various fields. Zhang et al.^31^ designed a multi-feature fusion faster Region-CNN (MF3 R-CNN) to solve the problem of insufficient images of soybean blades in complex scenes. However, manually combining images with Photoshop is labour-intensive and time-consuming.

The emergence of GANs has provided researchers with a new way of thinking^3^. Goodfellow et al. compared the recognition accuracy of enhancement methods such as Conditional Generative Adversarial Nets (CGAN)^32^, rotation and translation, and non-enhancement methods. The results proved that using GAN to generate images is superior to traditional data-enhancement methods. Wu et al.^33^ used a deep convolutional generative adversarial networks (DCGAN) to generate tomato leaf images and trained a GoogLeNet deep-learning network to recognise tomato leaf diseases. Hu et al.^34^ proposed a C-DCGAN to enhance tea disease samples and trained a VGG16 deep-learning network for disease recognition. However, this method converts noise into images, producing images of mediocre quality and containing much noise. Furthermore, this method is only suitable for generating more similar images from the same domain and cannot solve the imbalance between dataset categories. Another approach to image generation using GAN is image data enhancement using image pairs, which forms pairs of related images. Qu et al.^35^ proposed an enhanced Pix2Pix defogging network to solve the image-to-image translation problem and generate fog-free images without using a physical scattering model. Experimental results demonstrate that the model outperforms other methods. The method requires the geometric pairing of input images from two domains. However, in most cases, the one-to-one correspondence of image pairs is not satisfied.

Zhu et al.^36^ proposed an innovative CycleGAN, which can use unpaired images for image data enhancement. In the same year, Yi et al.^37^ proposed Dual GAN, which enables an image translator to be trained from two sets of unlabelled images from two domains, eliminating the need for paired data for image-to-image translation. Tian et al.^38^ used CycleGAN to address the issue of insufficient image data. To address the problem of insufficient image data due to the random occurrence of apple diseases, in addition to traditional image augmentation techniques, the CycleGAN deep-learning model was used to accomplish data augmentation. These methods effectively enriched the diversity of the training data and provided a solid foundation for training the detection model. Chen et al.^39^ proposed a framework combining YOLOv4 and CycleGAN to improve the quality of diamond pineapple surface-defect detection. The data enhancement of these image pairs all shares the same framework with two generators (GX2Y and GY2X) performing opposite transformations, which can be considered as double learning and two discriminators, DX and DY, to predict whether an image belongs to the domain or not. The generators (GX2Y, GY2X) transform the image from one domain to another. The discriminators (DX, DY) for each domain determine whether the image belongs to that domain or not.

### Generative adversarial network method based on self-attention mechanism

Traditional GANs have the problems of unstable training and poor generation quality. Although CycleGAN has made significant progress, it is difficult to intricately control the generated images owing to its unsupervised nature. Self-attention provides a solution to this problem. Pathak et al.^28^ were the first to train a generative adversarial network (GAN) with attention from scratch using a large-scale shelter image dataset, generating images with high PSNR and SSIM scores. Lu et al.^40^ proposed an image-translation method based on a self-attention mechanism. The main idea is to use a self-attention mechanism module in the generator network to expand the size of the feature map and enhance its description of the spatial structure around the central pixel. Compared with the original CycleGAN, the proposed method achieves excellent results, which fully demonstrates the strong data-augmentation ability of the proposed method. Li et al.^41^ proposed an SS-CycleGAN(Spatial and Semantic rationality CycleGAN) grayscale image coloring network and evaluated its performance on the natural color dataset and the flower dataset. Based on the cycle consistency adversarial network, SS-CycleGAN adds a self-attention mechanism to the generator and discriminator. The self-attention mechanism can guide the patch discriminator to pay attention to the semantic rationality of the object to be colored. Experimental results on a natural-color dataset and flower dataset verify the effectiveness of the proposed SS-CycleGAN. Aiming at the problem of the limited scale and insufficient diversity of maize leaf disease research dataset, Guo et al.^5^ proposed a maize-disease image-generation algorithm based on CycleGAN. Using the disease image-conversion method, practitioners can convert a healthy corn image to a disease crop image. The proposed method not only solves the limitations of existing maize disease datasets but also improves the accuracy of maize disease recognition in a small-sample maize-leaf disease-classification task. Additionally, Guowei et al.^42–44^ proposed a deep-learning model (PPLC-Net) consisting of an extended convolution, a multilevel attention mechanism, and a GAP layer. The model uses a novel meteorological data-enhancement method to expand the sample size and enhance the generalization and robustness of feature extraction. The feature-extraction network uses the zigzag expansion convolution with a variable expansion rate to expand the perceptual field of the convolution domain, which effectively solves the problem of insufficient spatial-information extraction. The lightweight CBAM attention mechanism is located in the middle layer of the feature-extraction network to enhance the information representation of the model. The GAP layer prevents the overfitting of the model by reducing the number and complexity of the network calculation parameters. Aiming at the problem of poor consistency between the enhanced sample and the original sample in the current data-augmentation methods, Siyuan et al.^45^ proposed a data-augmentation method to improve symplectic geometric reconstruction. This method expands a sufficient number of augmented samples while ensuring a high similarity with real samples.

So far, existing methods in crop disease image augmentation and colour distortion recovery suffer from training instability. It is challenging to converge CycleGAN to the equilibrium point using gradient training. Therefore, this paper proposes a new CycleGAN crop image data-augmentation method based on the self-attention mechanism to solve crop image colour distortion. The small dataset is insufficient to train CNNs.

## Methods

Deep-learning algorithms, such as CNNs and attention mechanisms, have successfully enhanced image data. Some achievements have been made in image data enhancement. This paper proposes a CIDE method based on SM-CycleGAN by combining the self-attention mechanism and the recurrent GAN. We design the general architecture of the model. In “CycleGAN principles” section introduces the principle of CycleGAN. In “SM-CycleGAN-based CIDE approach” section describes the CIDE method based on SM-CycleGAN, and the self-attention loss (SAL) function is introduced to reduce the long-tail problem between classes in “CIDE loss function” section.

### CycleGAN principles

CycleGAN is a variant of GAN networks using two transform networks to facilitate data transformations between domains. Dual GAN^35^ and Disco-GAN^46^ employ a similar idea that partially alleviates the need for data. Each transformation network is trained by a separate GAN network and its respective generator. The primary purpose of the discriminator is to distinguish the generated data from the real data.

#### Adversarial losses

Apply the adversarial loss to two mapping functions. For the mapping function *G*: *X* → *Y* and its identification ware *D*_*Y*_, denote the objective as

1 $$ L_{gan} \left( {G,D_{Y} ,X,Y} \right) = E_{y\sim pdata(y)} \left[ {\log D_{Y} \left( y \right)} \right] + E_{x\sim pdata(x)} \left[ {\log \left( {1 - D_{Y} \left( {G\left( x \right)} \right)} \right)} \right] $$

where *G* tries to generate an image *G*(*x*) that look similar to the image in domain *Y*, while *x* and *y* aim to distinguish between the panning sample *G*(*x*) and the real sample *Y*. A similar adversarial loss for the mapping function *F*: *Y* → *X* and its discriminator *D*_*X*_ as well: i.e. *L*_*gan*_ (*F*, *D*_*X*_, *Y*, *X*).

#### Loss of cyclic consistency

Adversarial training can learn mappings *G* and *F*, which produce outputs with the same distribution as the target domain *Y* and *X*, respectively. However, with sufficiently large capacity, the network can map the same set of input images to any random arrangement of images in the target domain, where either of the learned mappings can produce an output distribution that matches the target distribution. We further reduce the space of possible mapping functions by arguing that the learned mapping functions should be cyclically consistent using cyclic consistency loss to motivate this behaviour

2 $$ L_{cyc} \left( {G,F} \right) = E_{x\sim pdata\;\left( x \right)} \left[ {\left\| {F\left( {G\left( x \right)} \right) - x} \right\|_{1} } \right] + E_{y\sim pdata\;\left( y \right)} \left[ {\left\| {G\left( {F\left( x \right)} \right) - y} \right\|_{1} } \right] $$

#### Loss function of the original CycleGAN

The loss function of the original CycleGAN comprises three parts: generating adversarial loss for *X* to *Y*, generating adversarial loss for *Y* to *X*, and cyclic consistency loss function, denoted as

3 $$ L\left( {G,F,D_{X} ,D_{Y} } \right) = L_{gan} \left( {G,D_{Y} ,X,Y} \right) + L_{gan} \left( {F,D_{X} ,Y,X} \right) + \lambda L_{cyc} \left( {G,F} \right) $$

where *λ* is a weighting parameter to balance the importance of cyclic consistency and adversarial losses.

### SM-CycleGAN-based CIDE approach

**Figure 1:**
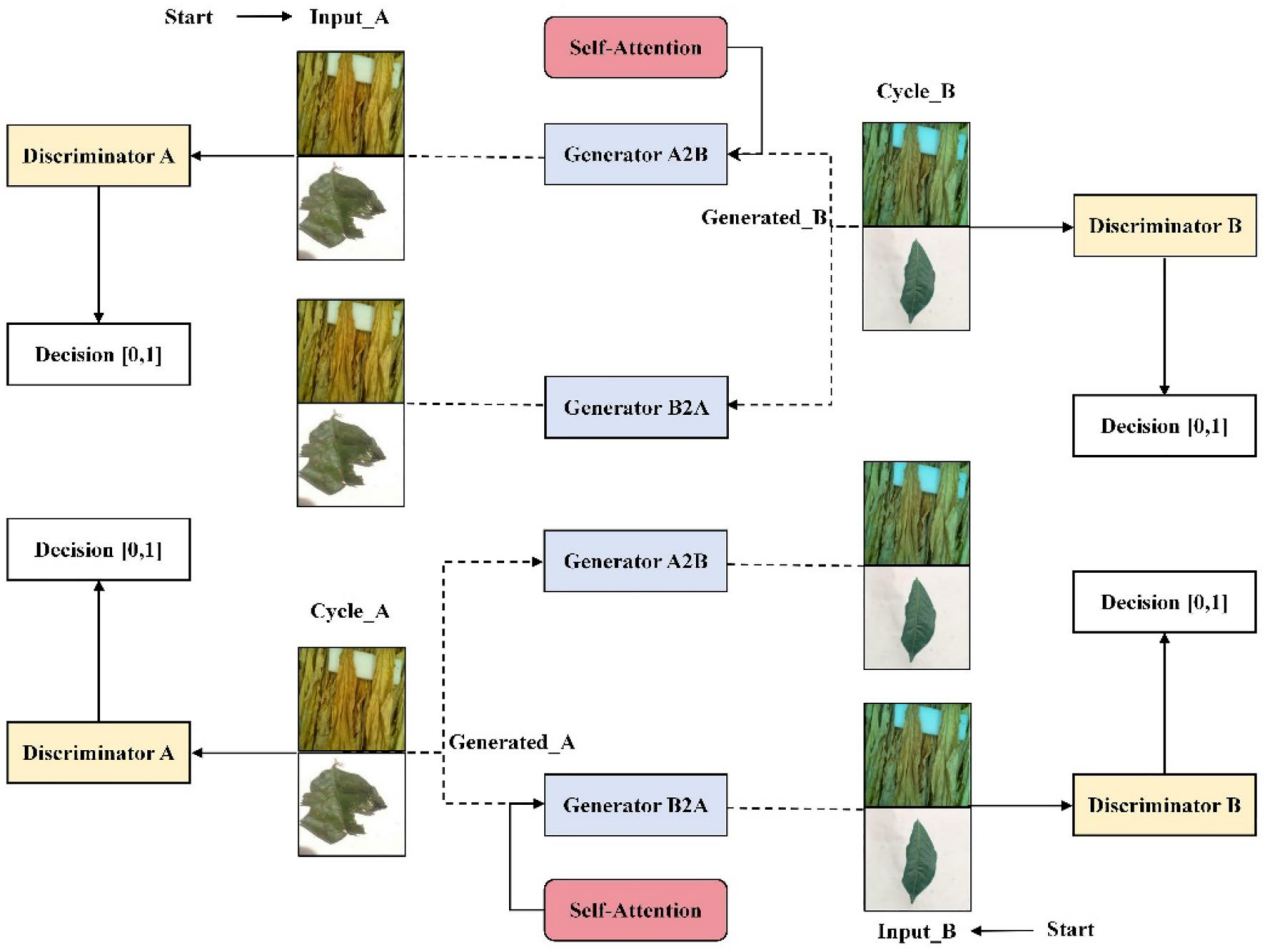
SM-CycleGAN crop image data-enhancement (CIDE) network.

The focus of this paper is to enhance crop image data. This paper constructs an improved crop image data-generation model by introducing the self-attention mechanism into CycleGAN, as shown in Fig. 1.

#### Self-attention mechanism

The attention mechanism used in generators and discriminators is the self-attention mechanism method^47^. This method highlights crucial features and reduces the influence of irrelevant information by learning the dependencies between different regions within the image. Self-attention mechanism localisation within the image is achieved by dynamically assigning weights to different regions, allowing the model to focus on the most critical regions of the image and better enabling the capture of global and local information. The self-attention mechanism generates a feature map the same size as the input image, identifying regions whose location is relevant to the label category. The computation of the feature map for the self-attention mechanism uses a global approach involving the entire feature map.

The self-attention mechanism operates on the feature maps obtained by convolution. The attention score is obtained by calculating the dot product between *Query* and *Key* and dividing it by $\sqrt {d_{k} }$ for scaling. This result is categorised using *SoftMax* to obtain the weight of each position. After calculating the matrix *Q*, *K*, and *V*, the output is obtained by

4 $$ Attention\left( {Q,K,V} \right) = soft\max \left( {\frac{{QK^{T} }}{{\sqrt {d_{k} } }}} \right)V $$

where *K*^*T*^ is the number of matrix columns of matrices *Q* and *K*.

#### Generating network structure

**Figure 2:**
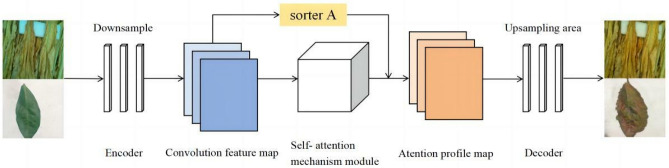
Structure of the generating network.

In the original CycleGAN model, the generator comprises an encoder, a converter and a decoder. The original CycleGAN used six ResBlock structures in its generator. With the inclusion of the self-attention mechanism, the six ResBlock structures in the converter are subdivided and added to the encoder and decoder of the generator network, respectively. Furthermore, the classifier A is included between the encoder and decoder of the generator. Figure 2 shows the generator structure based on the self-attention mechanism. In the augmented generator, as shown in Fig. 2, the input images are derived from the source domain *X* and the target domain *Y*. The generator’s encoder generates low-dimensional feature vectors. Classifier A determines whether the input image belongs to the source domain X by evaluating the feature maps from the input source and the target domain images after the generator encoded them. Furthermore, the self-attention technique allows a global pool to compute a weight value for each channel in the encoded feature map W. Using the principle of the self-attention mechanism, by multiplying and summing the weights of each channel with the encoded feature map, a feature map with the attention mechanism feature map is designed. Subsequently, this feature map with the attention mechanism is fed to the decoder of the generator, where it is up-sampled to recover its size to match the size of the input image. Classifier A in the generator is used as a binary classifier to recognise the feature maps generated from the input source and target domain images.

#### Discriminant network structure

**Figure 3:**
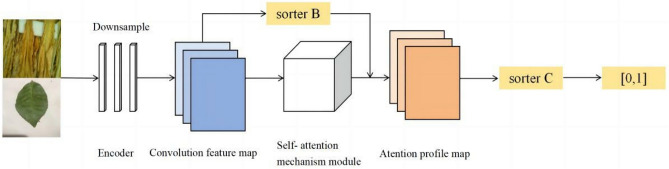
Structure of the discriminative network.

The input to the discriminator comprises real crop images and generated images. After down-sampling by the encoder, the input is convolved in one dimension, combined with bias vectors, and then passed through an S-shaped function to accomplish binary classification using probability values. The discriminator output value 1 indicates that the input crop image is recognised as real, whereas value 0 indicates that the generated crop image is classified. Figure 3 shows the network structure of the discriminator after integrating the self-attention mechanism module into the original model.

The discriminator integrates the self-attention mechanism module. As shown in Fig. 3, the discriminator receives input images from a generative image *G*(*X*) and a target domain image *Y*. The discriminator has two binary classifiers, B and C. Classifier B determines whether the input image is generative or from the target domain by processing the feature maps extracted from the input generative and target domain images encoded by discriminator *Ds*. The output of classifier B is the probability that the input image is generated. However, classifier C determines the same by processing the feature map of the attention mechanism generated by the self-attention mechanism. This feature map is obtained from the input generated and target domain images processed by the self-attention mechanism. The output of classifier C also indicates the probability that the input image is generated. Classifiers B and C aim to determine whether the input image originates from the generated or target domain images.

### CIDE loss function

#### SAL function

The SAL monitors the self-attention mechanism in a model to ensure that the model effectively captures fundamental relationships between elements as it learns sequence data. The goal is to encourage the model to focus on crucial elements during self-attention by assigning appropriate attention weights to better model dependencies in sequences.

5 $$ L_{{self{ - }attention}} = \sum\limits_{i = 1}^{n} {} \sum\limits_{j = 1}^{n} {similarity\left( {x_{i} ,x_{j} } \right)} \cdot \left( {1 - attention\left( {x_{i} ,x_{j} } \right)} \right) $$

where *x*_*i*_ and *x*_*j*_ are elements in the input sequence, and *n* is the sequence length. The goal of SAL is to minimise this loss to motivate the model to learn appropriate attention patterns to better model the relationships in sequence data and improve the performance of the model in various natural language processing and computer vision tasks.

#### CIDE loss function

6 $$ L\left( {G,F,DX,DY} \right) = L_{gan} \left( {G,DY,X,Y} \right) + L_{gan} \left( {G,DX,X,Y} \right) + \lambda L_{cyc} \left( {G,F} \right) + L_{{self{ - }attention}} $$

where *λ* is a weighting parameter to balance the importance of cyclic consistency and adversarial losses.

### Ethical statement

We promise to comply with the IUCN Policy Statement on Research Involving Species at Risk of Extinction and the Convention on the Trade in Endangered Species of Wild Fauna and Flora, and confirm that the research complies with relevant institutional, national and international norms and legislation.

## Isolated crop image online acquisition equipment

**Figure 4:**
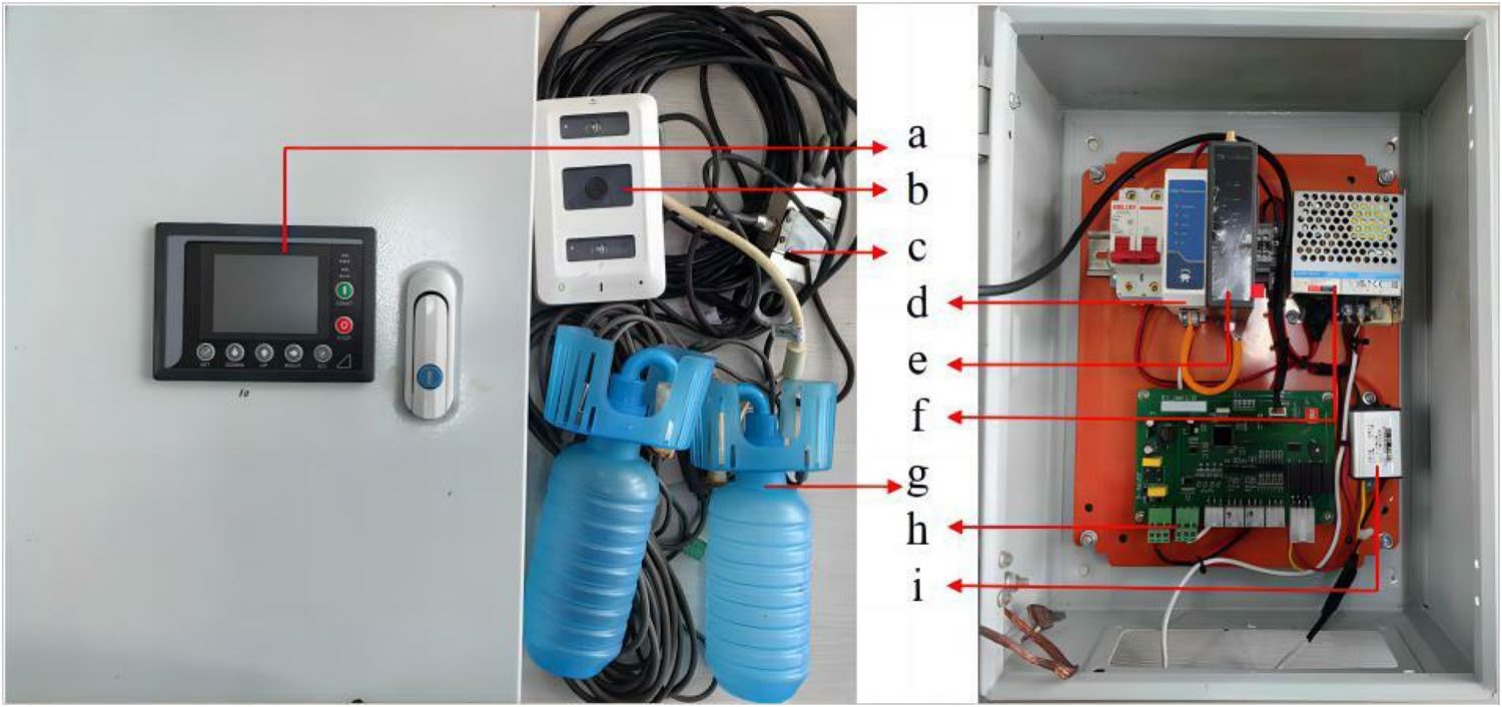
Real-time image integrated information acquisition equipment [(**a**) Real-time data display, (**b**) High-temperature and humidity-resistant camera, (**c**) Suspended weight sensor, (**d**) TCP data transmission module, (**e**) Dandelion router, (**f**) Switching power supply, (**g**) Wet and dry bulb temperature and humidity sensor, (**h**) Transformer, (**i**) Open-source development board].

The data used in this experiment were collected using a real-time crop image integrated information acquisition system (Fig. 4). The system comprises a high-temperature and high-humidity-resistant camera, a dandelion router, a Transmission Control Protocol (TCP) data transmission module, a switching power supply, and an open-source development board. The high-temperature and high-humidity camera is installed facing the crop. The dry and wet bulb temperature sensor, the S-type weight sensor and the display are connected to the Printed Circuit Board (PCB) development board. The image information acquisition system communicates with the tobacco-baking comprehensive information big data system through the TCP data transmission module and transmits the images and other information to the big data system for real-time display and storage.

## Experimentation and analysis

### Experimental environment

The experiments used an Ubuntu 20.04.2LTS operating system, a PyTorch deep-learning development framework, and Python as the development language. The Central Processing Unit (CPU) is Intel Core i7-9700F, and the Graphics Processing Unit (GPU) is NVIDIA GeForce RTX 3090Ti.

### Experimental process and assessment indicators

#### Experimental procedure

The following steps are taken to verify the effectiveness of the SM-CycleGAN model: (1) First, based on the tobacco roasting and tea disease image datasets, the quality of the generated images is evaluated using the structural similarity index (SSIM)^48^ and the peak signal-to-noise ratio (PSNR)^49^ as the objective evaluation indexes. (2) Compare and contrast the stability of the improved model through multiple experiments to verify its generative ability and stability. (3) Evaluate the performance of the SM-CycleGAN in this paper through ablation experiments and compare it with the original CycleGAN and other methods.

#### Assessment of indicators

To validate the performance of the SM-CycleGAN model in generating tea disease images and normal tobacco image augmentation, the PSNR and SSIM were used for quantitative comparison.

The PSNR is a metric used to compare the error between the corresponding pixels in the image of diseased tea leaves (normal tobacco) and the generated image (normal colour tobacco),

7 $$ PSNR = 10*\lg \left( {\frac{{MAX^{2} }}{MSE}} \right) $$

where *MAX* denotes the maximum possible value of an image pixel value, typically 255 in an 8-bit image. *MSE* denotes the mean squared error, which is the average of the squares of the differences of each pixel between the original and the compressed or distorted versions of an image or video.

The SSIM assesses the similarity between the data distribution of the normal diseased tea leaf blade (normal tobacco) image (*x*) and the generated diseased tea leaf blade (normal colour tobacco) image (*y*),

8 $$ SSIM = \frac{{\left( {2\mu_{x} \mu_{y} + C_{1} } \right)\left( {2\sigma_{xy} + C_{2} } \right)}}{{\left( {\mu_{x}^{2} + \mu_{y}^{2} + C_{1} } \right)\left( {\sigma_{x}^{2} + \sigma_{y}^{2} + C_{2} } \right)}} $$

where *x* denotes the original image. *y* denotes the distorted or processed image. *μ*_*x*_ and *μ*_*y*_ denote the luminance (average luminance) of *x* and *y*, respectively. *σ*_*x*_ and *σ*_*y*_ denote the variance of the luminance of *x* and *y*, respectively. *σ*_*xy*_ denotes the luminance covariance between *x* and *y*. C_1_ and C_2_ are constants to avoid zero values in the denominator; C_1_ = (*k*_1_*L*)^2^ and C_2_ = (*k*_2_*L*)^2^, where *L* denotes the dynamic range of the pixel value, typically 0–255 in 8-bit images.

#### Datasets

Two datasets were selected for this study: the tobacco roasting and tea disease image datasets. The tobacco leaf-baking image dataset is distributed in two categories: 206 normal and 206 colour-distorted tobacco leaf-baking images. The tea disease image dataset is distributed in five categories: 80 slices of normal and 80 slices of diseased tea images, totalling five categories, which is an imbalance. Each image has the same size as the input to the neural network (256 × 256 pixels) and is in the red–green–blue (RGB) colour space and JPG format. When the dataset is unbalanced between categories, the accuracy of deep learning in recognition will be severely affected. Therefore, considering the overfitting problem caused by too many parameters and too little data, SM-CycleGAN is used to generate images to balance or augment the dataset. Therefore, the healthy (non-normal colour) to diseased (normal colour) model is used to convert healthy (non-normal colour) leaves to leaves carrying disease (normal colour). The dataset is divided into a training set (including a validation set) and a test set (Table 1).

**Table 1:** Tobacco roasting and tea leaf disease image dataset settings.

	Tobacco-roasting image dataset			Tea disease image dataset		
	Data set	Training set	Test set	Data set	Training set	Test set
Quantity/pair	206	165	41	80	64	16
Percentage/%	100	80	20	100	80	20

#### Implementation details

SM-CycleGAN uses a CycleGAN (baseline) model pre-trained on ImageNet as the backbone network for crop image data enhancement, which is fine-tuned to optimize the performance of the model. The CNN extracts global features from 256 × 256 × 3 images as input for both generator and discriminator modules. These modules have different structures and parameters, with three MLPs mapping global, low-level, and high-level features to 256 × 256 × 3 using ReLU, LayerNorm, and Dropout respectively. We use 256 × 256 × 3 crop images as input to the image feature encoder with lsgan for SM-CycleGAN, CycleGAN, contrastive-unpaired-translation (CUT)^50^, AugCGAN^51^ and U-GAT-IT^52^; vanilla is used for Pix2Pix. During training we employ Adam optimizer with batch size set at 1; maximum number of epochs at 200; initial learning rate at 0.0002 to train each module from scratch. For SM-CycleGAN, CycleGAN, AugCGAN, U-GAT-IT weighting factors lambda_A,lambda_B,and lambda_identity are set at 10, 10, and 0.5, respectively. For Pix2Pix, the weighting factor lambda_L1 is set at 100; for CUT weighting factors lambda_GAN and lambda_NCE are set at 1 and 10 respectively.

### Tobacco-roasting image data analysis

#### Model stability analysis

**Figure 5:**
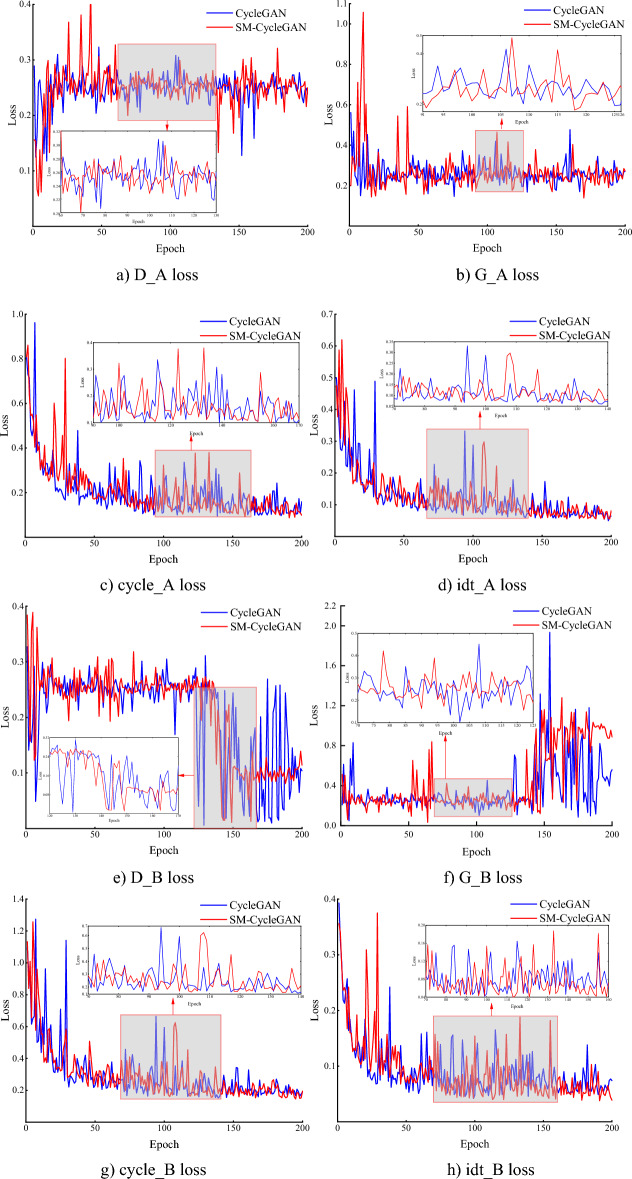
Comparison of loss function for tobacco-roasting image data recovery.

Figure 5 shows that on the tobacco-roasting image recovery dataset, although the loss function of SM-CycleGAN and the original CycleGAN is more oscillatory in the pre-training period, it shows a better convergence effect in the late training period. In particular, the D_B loss of SM-CycleGAN is more evident in the late training stage than in the original CycleGAN. This result indicates that the generator learns a better way to generate class A images after adding the attention mechanism, making it more challenging for the discriminator to distinguish between the generated and real images. For the G_B loss, it shows obvious oscillations at 140–180 epochs and then stabilises relative to the original CycleGAN, indicating that the generator becomes more effective in learning after adding the self-attention mechanism.

#### Comparative analysis of training time

**Figure 6:**
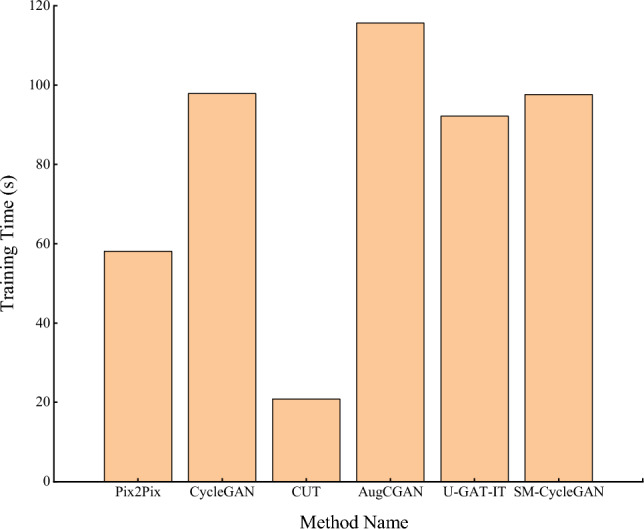
Comparative analysis of training time for tobacco-roasting images distortion recovery.

Figure 6 shows that on the tobacco-roasting image recovery dataset, SM-CycleGAN does not significantly increase the time for the entire training process compared to the original CycleGAN. This result indicates that even though the self-attention mechanism increases the computational cost of each training step, it does not significantly impact the overall training duration, and there is no additional loss of computational resources. And compared with AugCGAN and U-GAT-IT, the training time of SM-CycleGAN is closer.

#### Analysis of ablation experiments and comparative tests

1. Objective evaluation

**Figure 7:**
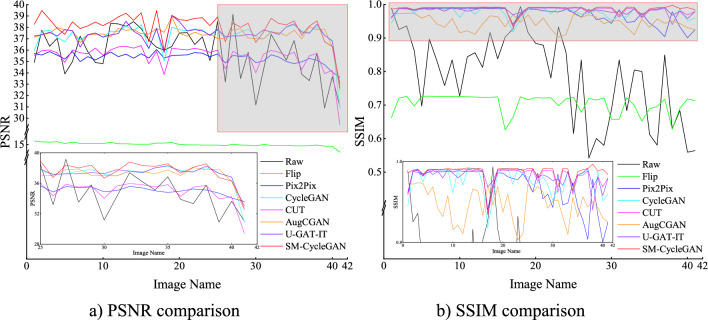
Comparison of evaluation metrics for the tobacco-roasting image recovery test set.

We conducted ablation experiments and comparison experiments. The experiments were conducted on non-normal-coloured tobacco images, and the quality of the generated images was compared. We compared the performance of SM-CycleGAN with offline data augmentation methods (Flip), Pix2Pix, Original CycleGAN (baseline method), CUT, AugCGAN and U-GAT-IT on the constructed tobacco leaf image dataset. Figure 7 shows the PSNR and SSIM comparisons of the validation sets of the seven methods for generating normal tobacco images, and Table 2 shows the average quality comparisons.

**Table 2:** Comparison of the quality of normal images of tobacco leaves generated using different methods.

	PSNR	SSIM
Raw	35.7980	0.7868
Flip	15.0173	0.7047
Pix2Pix	35.3236	0.9689
CycleGAN (Baseline)	37.2652	0.9757
CUT	35.4266	0.9820
AugCGAN	37.3533	0.9798
U-GAT-IT	37.3812	0.9825
SM-CycleGAN (Ours)	38.0592	0.9870

Table 2 shows that the SM-CycleGAN for generating normal tobacco images achieved higher PSNR and SSIM values than the original CycleGAN and other methods. For the normal tobacco images, SM-CycleGAN achieved a PSNR value of 38.0592 dB and an SSIM value of 0.9870, which is an improvement of 23.0435 dB and 0.2823, respectively, compared with Flip; an improvement of 2.7356 dB and 0.0181, respectively, compared with Pix2Pix; an improvement of 0.7940 dB and 0.0113, respectively, compared with CycleGAN; an improvement of 2.6326 dB and 0.0050, respectively, compared with CUT; an improvement of 0.7059 dB and 0.0072, respectively, compared with AugCGAN; an improvement of 0.6780 dB and 0.0045, respectively, compared with U-GAT-IT. These results demonstrate the effectiveness of the SM-CycleGAN in generating a normal tobacco image with an excellent resemblance to the real image, similar to the normal tobacco images with superior performance.

2. Subjective analysis

**Figure 8:**
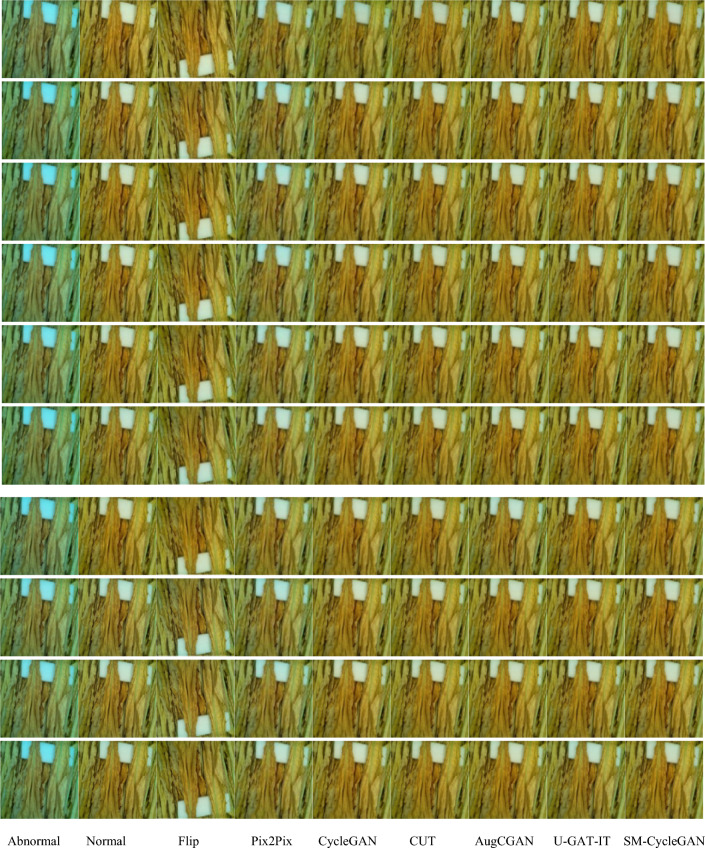
Comparison of tobacco-roasting image data enhancement by abnormal colour map, normal colour map, Flip, Pix2Pix, CycleGAN, CUT, AugCGAN, U-GAT-IT, SM-CycleGAN.

By the visual representation of the enhancement effect of non-normal-coloured tobacco image data, Fig. 8 shows that SM-CycleGAN shows better results in generating normal-coloured tobacco image data. Other methods show residual background interference and non-normal image colours in the generated images.

### Tea disease image data analysis

#### Model stability analysis

**Figure 9:**
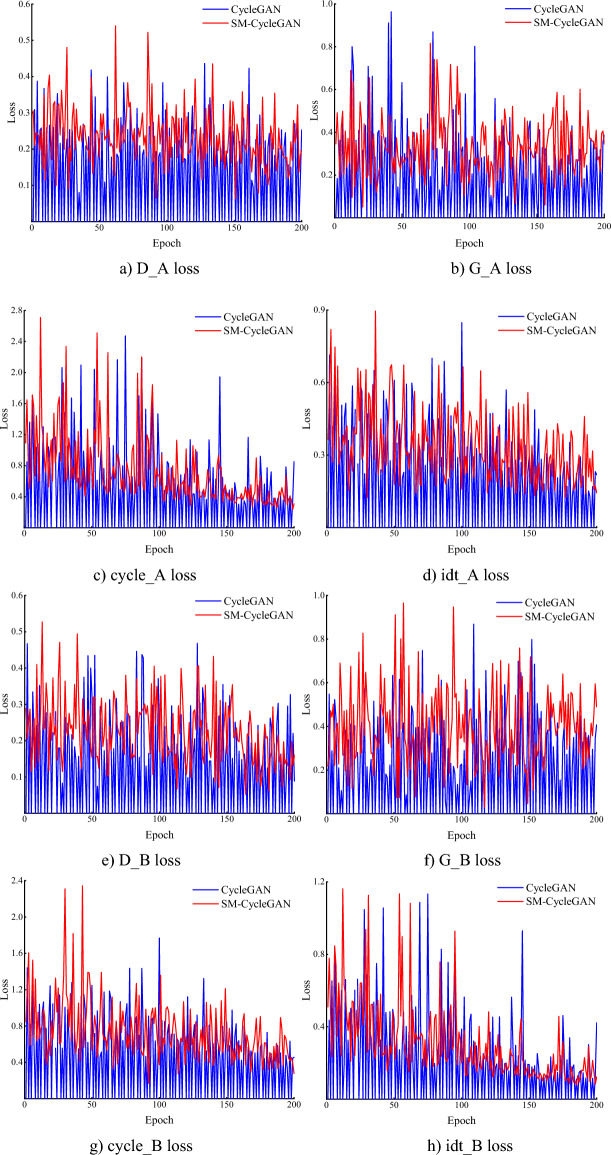
Comparison of amplification loss functions for tea disease images.

Figure 9 shows the loss function comparison analysis of the original CycleGAN and SM-CycleGAN models by tea disease image enhancement. When running the same epoch, the original CycleGAN did not converge, whereas the model gradually converged at the late training stage after adding the self-attention mechanism. The loss function oscillation of SM-CycleGAN is smoother than the CycleGAN, and the model converges faster, indicating that the generator and discriminator reach a more efficient state, which is more conducive to generating better image data.

#### Comparative analysis of training time

**Figure 10:**
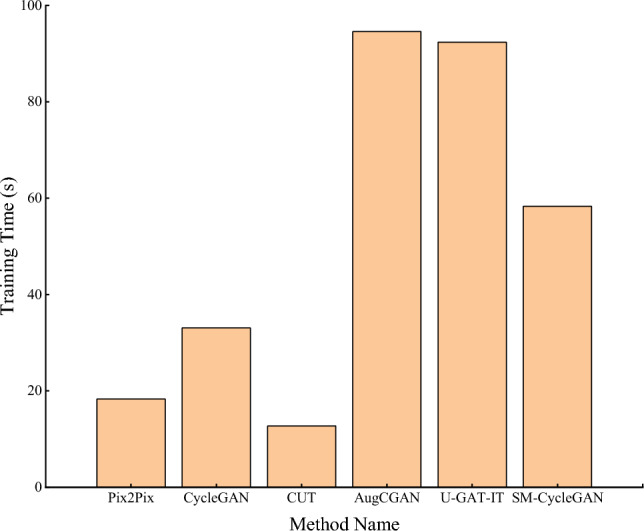
Comparative analysis of training time for tea disease images data enhancement.

Figure 10 shows that on the tea disease image dataset, SM-CycleGAN does not significantly increase the time of the whole training process compared with the original CycleGAN, indicating that even though the self-attention mechanism is introduced, the computational cost of each training step increases, but it does not have a great impact on the overall training time. However, SM-CycleGAN significantly reduces the time for the whole training process compared with AugCGAN and U-GAT-IT. Therefore, SM-CycleGAN can meet the training requirements while saving computing resources.

#### Analysis of ablation experiments and comparative tests

1. Objective evaluation

**Figure 11:**
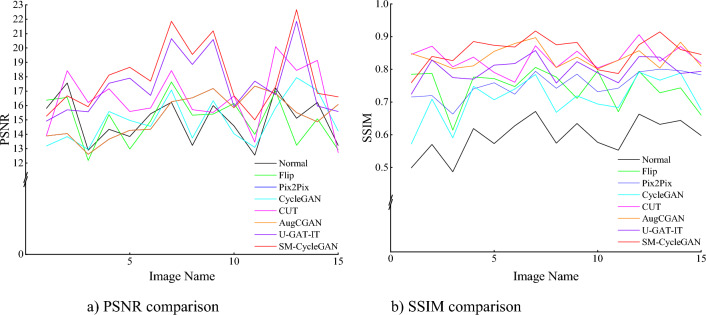
Comparison of evaluation metrics for the tea leaf disease image data-enhancement validation set.

We conducted ablation experiments and comparison experiments. The experiments were conducted to compare the quality of the generated images with tobacco colour distortion and tea leaf disease. We compared the performance of the proposed method with offline data augmentation methods (Flip), Pix2Pix, Original CycleGAN, CUT, AugCGAN and U-GAT-IT on the constructed tea disease image dataset. Figure 11 shows the PSNR and SSIM comparisons of the validation sets of the three methods for generating tea disease images, and Table 3 shows the average quality of the comparisons.

**Table 3:** Comparison of the quality of tea leaf disease images generated by different methods.

	PSNR	SSIM
Raw	14.9472	0.5948
Flip	15.0070	0.7445
Pix2Pix	15.2856	0.7527
CycleGAN (Baseline)	16.4872	0.8332
CUT	14.9609	0.7093
AugCGAN	16.7894	0.8364
U-GAT-IT	17.4944	0.7982
SM-CycleGAN (Ours)	17.9724	0.8539

Table 3 shows that the SM-CycleGAN for generating tea-leaf disease images achieved higher PSNR and SSIM values than the original CycleGAN and other methods. For the tea-leaf disease image, SM-CycleGAN achieved a PSNR value of 17.9724 dB and an SSIM value of 0.8539, which is an improvement of 2.9654 dB and 0.1094, respectively, compared with Flip; an improvement of 2.6868 dB and 0.1012, respectively, compared with Pix2Pix; an improvement of 1.3216 dB and 0.0207, respectively, compared with CycleGAN; an improvement of 3.0115 dB and 0.1446, respectively, compared with CUT; an improvement of 1.1830 dB and 0.0175, respectively, compared with AugCGAN; an improvement of 0.4780 dB and 0.0557, respectively, compared with U-GAT-IT. These results demonstrate the effectiveness of SM-CycleGAN in generating a tea-leaf disease image with an excellent resemblance to the real image, similar to the tea disease images with superior performance.

2. Subjective analysis

**Figure 12:**
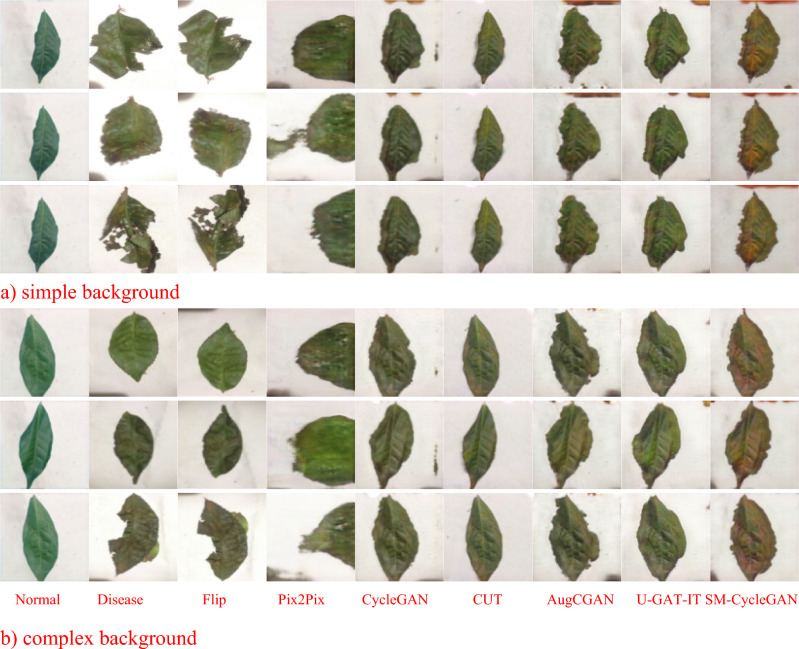
Study of the effect of generating images of tea leaf diseases using seven methods on different backgrounds.

Figure 12 shows the SM-CycleGAN performs the best for visualising tea leaf disease images generated by the three methods. In generating tea leaf disease images in different backgrounds, SM-CycleGAN avoids generating interfering images with disease characteristics in the background, thus producing more realistic diseased images. Pix2Pix performed the worst, with the tea leaf image losing crucial information but retaining the colour of the diseased image. Compared with the original CycleGAN and other methods, SM-CycleGAN shows some improvement regarding image clarity and interference information in the background of the image. However, it might still be challenging to generate disease colour feature information on complex backgrounds fully.

**Figure 13:**
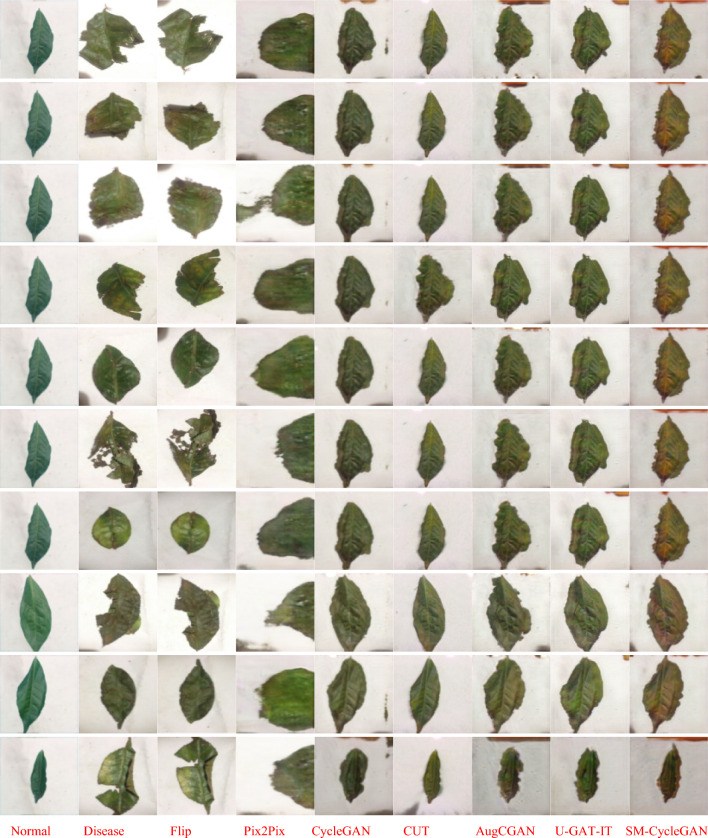
Comparison of tea leaf disease image data enhancement by normal map, disease map, Flip, Pix2Pix, CUT, CycleGAN, AugCGAN, U-GAT-IT, and SM-CycleGAN.

In Fig. 13, due to the intuitive representation of disease severity in severe tea leaf disease images where other methods show residual background interference in the generated images, SM-CycleGAN shows better results in generating tea leaf images severely affected by tea disease.

### Model robustness analysis

**Figure 14:**
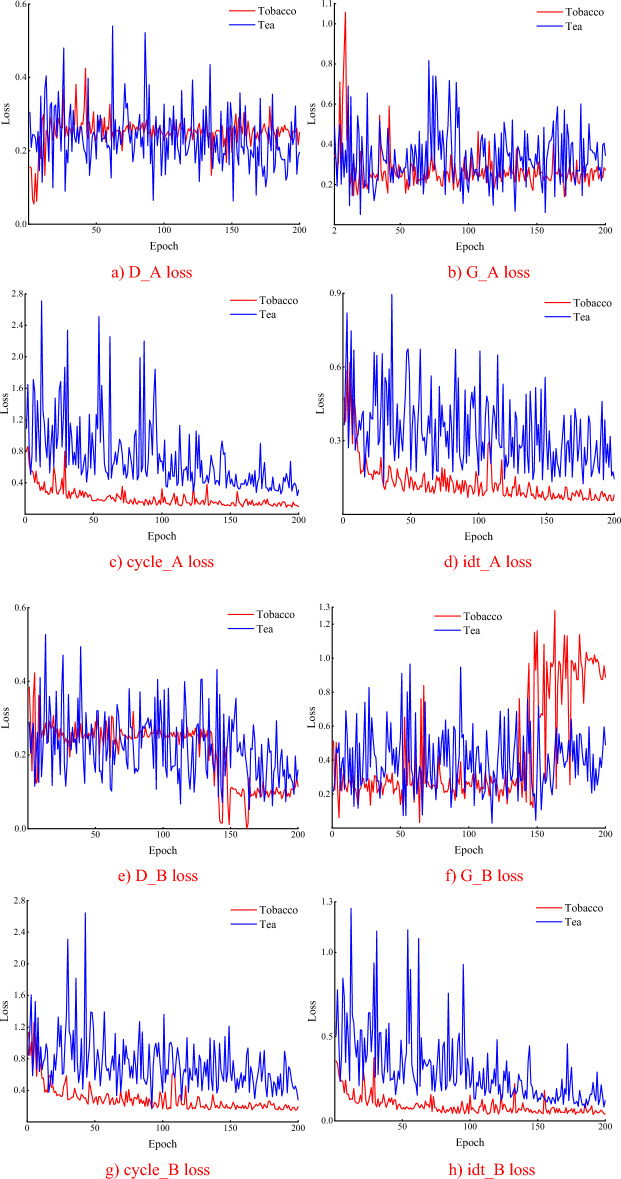
Comparison of SM-CycleGAN training loss function in tobacco leaf baking image dataset and tea leaf disease dataset.

Figure 14 demonstrates that when SM-CycleGAN is trained on the tobacco leaf baking image dataset and the tea leaf disease dataset, the loss function of the former converges at a lower value compared to that of the latter. Notably, all four losses (cycle_A loss, cycle_B loss, idt_A loss, and idt_B loss) exhibit rapid convergence with their values gradually approaching zero. A smaller loss function indicates greater model stability and robustness; thus, the SM-CycleGAN model demonstrates superior robustness in handling tobacco baking image datasets. This can be attributed to the larger volume of image data available in the tobacco leaf baking image dataset compared to that in the tea disease image dataset. The abundance of data enables more comprehensive and accurate information for effective learning of genuine patterns within the data while mitigating overfitting.

## Conclusion

We propose a crop image data enhancement method based on SM-CycleGAN to realize the restoration and expansion of the crop dataset. In order to test the performance of the SM-CycleGAN model, we use the original CycleGAN and other six methods to conduct ablation and comparison experiments on the tobacco leaf baking image data set and the tea leaf disease image data set. The experimental results show that, taking the tobacco leaf baking image as an example, compared with the tobacco leaf normal image quality, SM-CycleGAN improves the PSNR and SSIM by 2.13%and 1.16%respectively compared with the original CycleGAN. Taking the tea image as an example, compared with the tea leaf disease images, the PSNR and SSIM of SM-CycleGAN are improved by 3.55% and 2.48%. By comparing the quality of generated images of different crops, SM-CycleGAN is better than other methods in PSNR and SSIM indicators of generated images. In summary, SM-CycleGAN effectively expanded the datasets, increasing the available training samples and achieving sustainable development in agricultural disease and baking data. The results can be applied to improve the early identification of crop leaf diseases and the classification of crop diseases in the baking stage, which can ultimately help to improve the efficiency of agricultural production.

Although our method can yield good enhanced results, we only collected two crop data sets for experimental analysis due to time constraints. In the future, we will collect image data of other types of crops and diseases for training and testing to enhance the generalization ability of SM-CycleGAN.

## Data Availability

Data is provided within the manuscript information files. The tobacco image dataset in this paper is created by using the images collected from the tobacco curing factory in Bozhou district, Zunyi City, Guizhou Province, published in https://blog.csdn.net/ld1314_/article/details/137572185; and the tea image dataset in this paper is created using images from the linked datasets below: https://blog.csdn.net/qq_40840797/article/details/131837877.

## References

[1] Hu G, Li S, Wan M (2021). Semantic segmentation of tea geometrid in natural scene images using discriminative pyramid network. Appl. Soft Comput..

[2] Gao Y, Cao Z, Cai W (2023). Apple leaf disease identification in complex background based on BAM-Net. Agronomy.

[3] Goodfellow I, Pouget-Abadie J, Mirza M (2014). Generative adversarial nets. Adv. Neural Inf. Process. Syst..

[4] Achddou R, Gousseau Y, Ladjal S (2023). Fully synthetic training for image restoration tasks. Comput. Vis. Image Underst..

[5] Guo H, Li M, Hou R (2023). Sample expansion and classification model of maize leaf diseases based on the self-attention CycleGAN. Sustainability.

[6] Van Marrewijk BM, Polder G, Kootstra G (2022). Investigation of the added value of CycleGAN on the plant pathology dataset. IFAC-PapersOnLine.

[7] Almasoud AS, Abdelmaboud A, Eisa TAE (2022). Artificial intelligence-based fusion model for paddy leaf disease detection and classification. Comput. Mater. Contin..

[8] Al-Wesabi FN, Albraikan AA, Hilal AM (2022). Artificial intelligence enabled apple leaf disease classification for precision agriculture. Comput. Mater. Contin..

[9] Chen, X. *et al.* Underwater image enhancement using CycleGAN. In *NCIT 2022; Proceedings of International Conference on Networks, Communications and Information Technology, Virtual*, 1–5 (2022).

[10] Chen Y, Pan J, Wu Q (2023). Apple leaf disease identification via improved CycleGAN and convolutional neural network. Soft Comput..

[11] Liu W, Zhai Y, Xia Y (2023). Tomato leaf disease identification method based on improved YOLOX. Agronomy.

[12] Xiao D, Zeng R, Liu Y (2022). Citrus greening disease recognition algorithm based on classification network using TRL-GAN. Comput. Electron. Agric..

[13] Sun C, Huang C, Zhang H (2022). Individual tree crown segmentation and crown width extraction from a heightmap derived from aerial laser scanning data using a deep learning framework. Front. Plant Sci..

[14] Zeng W, Li M (2020). Crop leaf disease recognition based on self-attention convolutional neural network. Comput. Electron. Agric..

[15] Qian X, Zhang C, Chen L (2022). Deep learning-based identification of maize leaf diseases is improved by an attention mechanism: Self-attention. Front. Plant Sci..

[16] Stephen A, Punitha A, Chandrasekar A (2023). Designing self attention-based ResNet architecture for rice leaf disease classification. Neural Comput. Appl..

[17] Hu Y, Deng X, Lan Y (2023). Detection of rice pests based on self-attention mechanism and multi-scale feature fusion. Insects.

[18] Meng Y, Yuan W, Aktilek EU (2023). Fine hyperspectral classification of rice varieties based on self-attention mechanism. Ecol. Inf..

[19] He Z, Chen G, Zhang Y (2023). Pyramid feature fusion through shifted window self-attention for tobacco leaf classification. Expert Syst. Appl..

[20] Bao W, Zhu Z, Hu G (2023). UAV remote sensing detection of tea leaf blight based on DDMA-YOLO. Comput. Electron. Agric..

[21] Bao W, Yang X, Liang D (2021). Lightweight convolutional neural network model for field wheat ear disease identification. Comput. Electron. Agric..

[22] Arun Pandian, J., Geetharamani, G. & Annette, B. Data augmentation on plant leaf disease image dataset using image manipulation and deep learning techniques. In *2019 IEEE 9th International Conference on Advanced Computing (IACC)*, 199–204 (2019). 10.1109/IACC48062.2019.8971580.

[23] Odabas MS, Şenyer N, Kurt D (2023). Determination of quality grade of tobacco leaf by image processing on correlated color temperature. Concurr. Comput. Pract. Exp..

[24] Lu J, Hu J, Zhao G (2017). An in-field automatic wheat disease diagnosis system. Comput. Electron. Agric..

[25] Ma J, Du K, Zheng F (2018). A recognition method for cucumber diseases using leaf symptom images based on deep convolutional neural network. Comput. Electron. Agric..

[26] Rasti S, Bleakley CJ, Silvestre GC (2023). Assessment of deep learning methods for classification of cereal crop growth stage pre and post canopy closure. J. Electron. Imaging.

[27] Rasti S (2021). Crop growth stage estimation prior to canopy closure using deep learning algorithms. Neural Comput. Appl..

[28] Rasti S, Bleakley C, Holden NM (2021). A survey of high resolution image processing techniques for cereal crop growth monitoring. Inf. Process. Agric..

[29] Chen Y, Pan J, Wu Q (2023). Apple leaf disease identification via improved CycleGAN and convolutional neural network. Soft Comput..

[30] Kukačka, J., Golkov, V. & Cremers, D. Regularization for deep learning: A taxonomy. arXiv preprint arXiv:1710.10686 (2017). 10.48550/arXiv.1710.10686.

[31] Zhang K, Wu Q, Chen Y (2021). Detecting soybean leaf disease from synthetic image using multi-feature fusion faster R-CNN. Comput. Electron. Agric..

[32] Mirza, M. & Osindero, S. Conditional generative adversarial nets. arXiv preprint arXiv:1411.1784 (2014). 10.48550/arXiv.1411.1784.

[33] Wu Q, Chen Y, Meng J (2020). DCGAN-based data augmentation for tomato leaf disease identification. IEEE Access.

[34] Hu G, Wu H, Zhang Y (2019). A low shot learning method for tea leaf’s disease identification. Comput. Electron. Agric..

[35] Qu, Y., Chen, Y., Huang, J. & Xie, Y. Enhanced Pix2pix Dehazing Network. In *2019 IEEE/CVF Conference on Computer Vision and Pattern Recognition (CVPR)*, 8152–8160 (2019). 10.1109/CVPR.2019.00835.

[36] Zhu, J.-Y., Park, T., Isola, P. & Efros, A. A. Unpaired image-to-image translation using cycle-consistent adversarial networks. In *2017 IEEE International Conference on Computer Vision (ICCV)*, 2242–2251 (2017). 10.1109/ICCV.2017.244.

[37] Yi, Z., Zhang, H., Tan, P. & Gong, M. DualGAN: Unsupervised dual learning for image-to-image translation. In *2017 IEEE International Conference on Computer Vision (ICCV)*, 2868–2876 (2017). 10.1109/ICCV.2017.310.

[38] Tian Y, Yang G, Wang Z (2019). Detection of apple lesions in orchards based on deep learning methods of CycleGAN and YOLOV3-dense. J. Sens..

[39] Chen SH, Lai YW, Kuo CL (2022). A surface defect detection system for golden diamond pineapple based on CycleGAN and YOLOv4. J. King Saud Univ. Comput. Inf. Sci..

[40] Lu, Y., Liu, J., Zhao, X. *et al*. Image translation with attention mechanism based on generative adversarial networks. In *IEEE INFOCOM 2020—IEEE Conference on Computer Communications Workshops (INFOCOM WKSHPS)*, 364–369 (2020). 10.1109/INFOCOMWKSHPS50562.2020.9162836.

[41] Li B, Lu Y, Pang W (2023). Image colorization using CycleGAN with semantic and spatial rationality. Multimed. Tools Appl..

[42] Dai G, Fan J, Tian Z (2023). PPLC-Net: Neural network-based plant disease identification model supported by weather data augmentation and multi-level attention mechanism. J. King Saud Univ. Comput. Inf. Sci..

[43] Dai G, Tian Z, Fan J (2024). DFN-PSAN: Multi-level deep information feature fusion extraction network for interpretable plant disease classification. Comput. Electron. Agric..

[44] Dai G, Fan J, Dewi C (2023). ITF-WPI: Image and text based cross-modal feature fusion model for wolfberry pest recognition. Comput. Electron. Agric..

[45] Liu S, Yin J, Hao M (2024). Fault diagnosis study of hydraulic pump based on improved symplectic geometry reconstruction data enhancement method. Adv. Eng. Inform..

[46] Kim, T., Cha, M., Kim, H. *et al*. Learning to discover cross-domain relations with generative adversarial networks. In *International Conference on Machine Learning*, 1857–1865 (2017). 10.48550/arXiv.1703.05192.

[47] Vaswani, A., Shazeer, N., Parmar, N. *et al*. Attention is all you need. arXiv (2017). 10.48550/arXiv.1706.03762.

[48] Wang Z, Bovik AC, Sheikh HR (2004). Image quality assessment: From error visibility to structural similarity. IEEE Trans Image Process..

[49] Sheikh HR, Sabir MF (2006). A statistical evaluation of recent full reference image quality assessment algorithms. IEEE Trans Image Process..

[50] Park, T., Efros, A. A., Zhang, R. & Zhu, J. Contrastive learning for unpaired image-to-image translation. In *European Conference on Computer Vision* (2020).

[51] Almahairi, A., Rajeshwar, S., Sordoni, A., Bachman, P. & Courville, A. Augmented CycleGAN: Learning many-to-many mappings from unpaired data. In *International Conference on Machine Learning* (2018). 10.48550/arXiv.1802.10151.

[52] Kim, J., Kim, M., Kang, H. & Lee, K. U-GAT-IT: Unsupervised generative attentional networks with adaptive layer-instance normalization for image-to-image translation. arXiv abs/1907.10830. 10.3390/s23156858 PMC10422294 37571641

